# Ecology and evolution of facilitation among symbionts

**DOI:** 10.1038/s41467-018-06779-w

**Published:** 2018-11-19

**Authors:** Flore Zélé, Sara Magalhães, Sonia Kéfi, Alison B. Duncan

**Affiliations:** 1 cE3c: Centre for Ecology, Evolution, and Environmental Changes, Faculty of Sciences, University of Lisbon, Edifício C2, piso-3, 1749-016 Lisboa, Portugal; 2 ISEM, Université de Montpellier, CNRS, EPHE, IRD, Montpellier, Cedex 05 France

## Abstract

Facilitation occurs when one species positively impacts the fitness of another, and has predominantly been studied in free-living species like plants. Facilitation can also occur among symbiont (mutualistic or parasitic) species or strains, but equivalent studies are scarce. To advance an integrated view of the effect of facilitation on symbiont ecology and evolution, we review empirical evidence and their underlying mechanisms, explore the factors favouring its emergence, and discuss its consequences for virulence and transmission. We argue that the facilitation concept can improve understanding of the evolutionary forces shaping symbiont communities and their effects on hosts.

## Abstract (web summary)

Facilitation is a well-known ecological interaction among free-living species, but symbionts residing in or on a host can also positively affect other symbiont species. Here, the authors review examples of facilitation among symbionts, revealing how facilitation theory can improve understanding of these interactions.

## Introduction

**Fig. 1:**
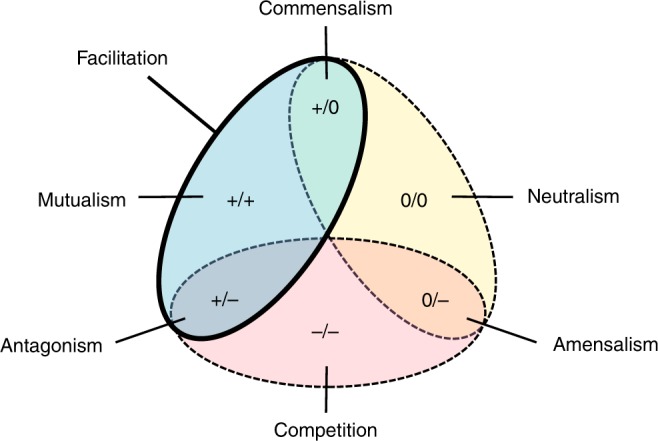
Diagram showing the different types of ecological interactions. 0: no effect; –: negative effect; +: positive effect. Facilitation includes mutualistic, commensal and antagonistic interactions

It is now widely accepted that interacting species can positively impact one another^1–5^. Facilitation (Box 1) is one of the broadest terms referring to these positive interactions (Fig. 1). Its history is anchored in that of plant–plant interactions, although its realm has recently been extended to include other taxa^1^. Studies to date generally document its occurrence, and the ecological consequences of facilitation for individuals, species and ecosystems. The evolutionary causes and consequences of these interactions, though tackled using modelling approaches^6^ and phylogenetic analyses^7^, are still poorly assessed via contemporary evolution studies^2^. Despite the ubiquity of organisms that live within or upon others (endo or ecto-symbionts, hereafter ‘symbionts’ for brevity; Box 1), facilitation between symbionts has been largely overlooked. Indeed, individual hosts are often colonised by multiple symbionts, which can have positive, negative or neutral effects on one another (Fig. 1), independently of their effect on the host (e.g. either parasitic, commensal or mutualistic). Symbiont–symbiont competition has been shown both empirically and theoretically to have diverse effects on symbiont ecology and evolution^8^, but corresponding studies about facilitation in multiple infections (Box 1) are lacking. The host-symbiont literature contains many examples of symbiont–symbiont interactions compatible with facilitation (see Supplementary Table 1), though the interactions are rarely identified as such and are not unified into a common body of work. Moreover, a number of these studies investigate the evolutionary outcomes of facilitation^9,10^, which may be relevant for the interpretation of ecological patterns observed in both symbiotic and free-living systems.

Several reviews state that facilitative interactions have been relatively neglected in ecological theory, despite abundant empirical evidence for their occurrence in natural populations and indication of their importance for community functioning and stability^1,2,5^. Here, we argue that facilitation has been particularly overlooked in the symbiont literature^4^. The aim of this review is to highlight that integrating approaches used to study facilitation in free-living organisms with studies of symbionts will be highly informative and beneficial for both fields of research. On the one hand, placing symbiont–symbiont interactions in the context of facilitation should increase our understanding about infection outcomes. On the other hand, because it is easier to study evolution in symbionts than in most free-living organisms (given their shorter generation time), studies of symbiont–symbiont facilitation may guide predictions about how positive interactions between species could shape evolution in free-living communities.

First, we outline the different mechanisms of symbiont–symbiont facilitation. Next, we investigate the ecological and evolutionary conditions favouring the occurrence and maintenance of facilitation between symbiotic organisms. Finally, we discuss the ecological and evolutionary consequences of facilitation, and suggest future research avenues. Throughout, we highlight parallels with free-living organisms.

### Box 1 Glossary

**Co-transmission:** two or more symbionts are transmitted together, sometimes packaged together in the same protein case.

**Facilitation:** any interaction where the action of one symbiont has a beneficial effect on another. This includes mutualistic interactions where both the facilitated and facilitator benefit (+/+), those which are commensal (+/0) when the effects of the facilitated on the facilitator are neutral as well as those which are antagonistic (+/−) when the facilitated negatively impact the facilitator (Fig. 1). Note that this concept partially overlaps with that of mutualism, ecological engineering and niche construction.

**Multiple infection:** the presence of more than one symbiont (strain or species) circulating in an individual or population.

**Symbiont:** As defined by Anton de Bary (1879): ‘the living together of unlike organisms’, we use this term to refer to any organism residing within or on hosts, encompassing all species along the mutualist–parasite continuum (i.e. they can be mutualists, commensalists or parasites of the host).

**Syntrophy:** nutritional relationship between two organisms that combine their metabolic abilities to use a substrate that they could not use otherwise. A special case of syntrophy is cross-feeding, in which two organisms feed on the waste products of each other.

**Transmission:** the passage of a symbiont from one host to another.

**Transmission mode:** the relationship between hosts among which symbionts are transmitted. Vertical transmission occurs from parent to offspring; horizontal transmission from infected to uninfected hosts, via non-hereditary mechanisms, often the environment; and mixed transmission is a combination of vertical and horizontal transmission.

**Transmission route:** the means that symbionts use to pass from one host to another (e.g. body fluids, a vector, spores in the environment). ‘Direct contact transmission’ is through host-to-host contact (e.g. coughing), whereas indirect transmission occurs via a vector (i.e. the environment or another host).

**Virulence:** symbiont-induced reduction in host fitness.

## Mechanisms of facilitation

**Fig. 2:**
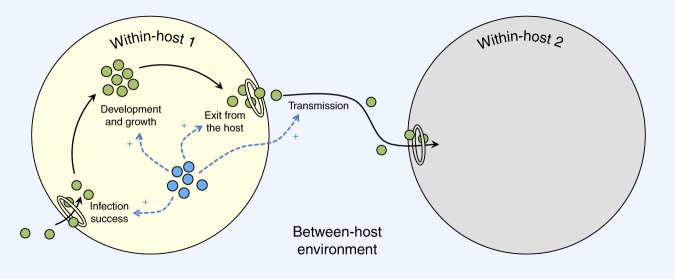
Facilitation between symbionts can occur within or between hosts. Small blue circles: facilitating symbionts; small green circles: facilitated symbionts; big yellow circle: co-infected host; big grey circle: uninfected host; rings: entry/exit point of the host; blue dashed arrows: facilitation; black solid arrows: transition between life cycle stages of the facilitated symbiont. Traits facilitated are categorised as ‘development’ (i.e. symbiont growth or differentiation) in the within-host environment, ‘infection success’ (i.e. host entry or establishment), ‘exit from the host’ at the interface between the within- and between-host environment, and ‘transmission’ in the between-host environment. Note that the diagram does not consider the symmetry of the interaction (i.e. the effect of the facilitated symbiont on the facilitator is not represented)

**Fig. 3:**
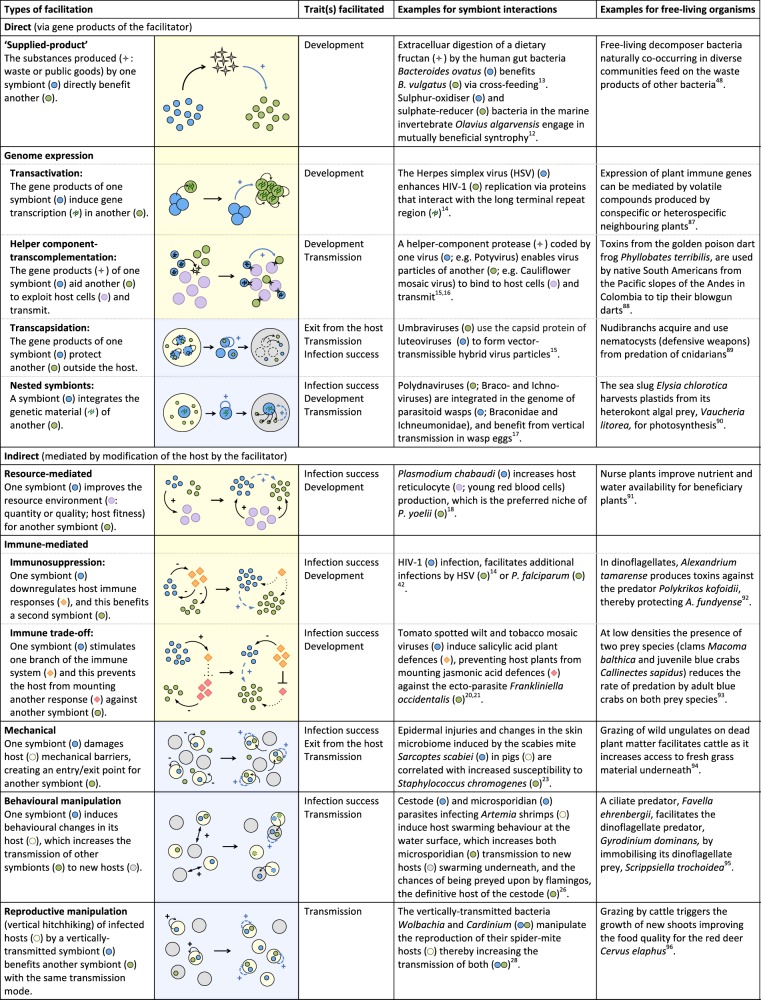
Mechanisms underlying symbiont facilitation and parallels with free-living organisms. Blue circles: facilitating symbionts; green circles: facilitated symbionts; yellow shading: within-host environment; blue shading: between-host environment; grey or yellow circles: host; purple circles: resource; diamonds: host immune response; blue arrows: facilitation (solid: direct; dashed: indirect); black arrows: other types of effects (solid: enabled; dotted: cancelled). Traits facilitated are categorised as ‘development’ (i.e. symbiont’s growth or differentiation) in the within-host environment, ‘infection success’ (i.e. host entry or establishment) and ‘exit from the host’ at the interface between the within- and between-host environment, and ‘transmission’ in the between-host environment

Symbionts can facilitate each other either directly (independently of the host) or indirectly (via host manipulation) and facilitation can occur both within- and between-hosts (Fig. 2). Overall, the mechanisms of facilitation between symbionts are similar to those found between free-living organisms (summarised in Fig. 3, along with some chosen examples; see also ref. ^11^, and Supplementary Table 1 for more examples of facilitation between symbionts).

### Direct facilitation

Some symbionts directly facilitate the growth or reproduction of others, by producing substances aiding them to exploit the host (i.e. ‘supplied-product’ facilitation)^12,13^. Direct facilitation can occur when a symbiont facilitates another by affecting its gene expression (i.e. transactivation)^14^ or by providing essential gene products such as in the case of transcapsidation^15^ or helper component-transcomplementation^15,16^. Direct facilitation can also arise when exogenous genetic material from one symbiont becomes integrated in another (i.e. nested symbionts)^17^.

### Indirect facilitation

Within-host indirect facilitation can be mediated by the modification of host resources used by symbionts^18^, or by improving host fitness in ways that benefit other symbionts. For instance, by increasing host longevity, a symbiont can reduce the survival cost of infection by another parasite^19^, which, in turn enhances the probability that the latter completes its development within the host.

Indirect facilitation also occurs via the host immune system. This can be brought about by immune-evasion strategies such as immunosuppression, which might be advantageous for other symbionts within the host^14^, or via immunological trade-offs, whereby a host is unable to simultaneously mount immune responses against different symbionts^20–22^. Furthermore, symbionts can facilitate host entry or exit of another^4^ via epidermal injuries^23^ or through the symptoms of infection^24^.

Finally, behavioural or reproductive manipulation of the host by a symbiont can facilitate the transmission (Box 1) of other symbionts (e.g. ‘hitch-hiking’)^25^. This might occur between horizontally transmitted symbionts with complex life cycles that share both intermediate and definitive hosts^25^, as well as between symbionts with different transmission routes^26^ (Box 1). Vertically transmitted reproductive manipulators that increase the proportion of infected female offspring in the population^27^ can also facilitate the transmission of other vertically transmitted symbionts (i.e. via synergy or hitchhiking)^27,28^.

## Multiple mechanisms and multiple effects

Facilitation is often not easily attributed to a single mechanism. For example, HIV-1 triggers lymphocyte activation, and activated lymphocytes are the preferred resource of the human cytomegalovirus^29^. Hence, facilitation is immune-mediated from the facilitator perspective, but resource-mediated for the facilitated. Moreover, several facilitating mechanisms can operate simultaneously. For instance, the direct facilitation of polydnavirus transmission by parasitoid wasps described in Fig. 3 is accompanied by an indirect facilitation by the virus for wasps, via silencing of the host immune system^17^. Importantly, many (if not all) multiple infections entail some degree of competition between symbionts. This means that interactions between the same two organisms can be both facilitative and competitive, changing at different life-stages or in different environments^11^. Moreover, facilitation and competition can occur over the same resource/common good (Fig. 3), or facilitation might occur for one trait and competition for another^30^. For example, simultaneous infection of rabbits by two helminth species increases the density but decreases the fecundity of one symbiont, while having the opposite effect on the other^31^. Whether this can be considered as facilitation is debatable, and would ultimately require measuring the transmission of each parasite to new hosts, to ascertain whether the net direction of the interaction is negative or positive.

Similarly, it is not always clear whether competition or facilitation is the dominant interaction between a facilitator and a facilitated individual in plant species pairs^32^ (but see ref. ^33^). Consequently, the net effect of a facilitator on a facilitated results from unequal negative and positive effects, and some consider that the term ‘facilitation’ should be reserved for cases where positive effects dominate^11^. The interplay between competition and facilitation forms the basis of a classical prediction in the ecological literature, the stress-gradient hypothesis^34^: it states that positive interactions should be more frequent under more stressful environmental conditions, whereas negative interactions are expected to be more frequent under benign conditions^34^. A recent meta-analysis confirmed that competitive interactions declined with increasing stress between 727 pairs of plant species^35^. Whether this holds for pairs of symbiont species remains to be tested.

Another issue arises from potential correlations between symbiont life-history traits at the within/between-host level^36^. Indeed, a facilitator causing higher within-host symbiont growth might be assumed to also increase transmission (as these traits are often positively correlated and linked to fitness^37^). However, this does not always hold true. For instance, in *Culex pipiens* mosquitoes, *Wolbachia* increases *Plasmodium relictum* infection success and within-host growth, but not the number of transmissible stages^38^. Moreover, by increasing symbiont load, the presence of a facilitator can induce increased virulence (Box 1), which, in turn, might reduce transmission through a shortened infection duration^37^. Indeed, mathematical models parameterised from laboratory data show that facilitation measured as higher within-host growth does not always result in more severe epidemics^18^. Alternatively, the within-host effects of one symbiont (e.g. increased host fecundity leading to larger host populations) might facilitate another in the between-host environment (e.g. because there are more available hosts).

As highlighted by Alizon and Michalakis^36^, a fitness-based approach, which encompasses the entire symbiont life cycle, should be adopted when studying facilitation. This provides an estimate of total symbiont fitness rather than individual fitness components, and accounts for potential pleiotropic effects of facilitation on different traits (or hosts for multi-host symbionts^26^). Although it can be complicated to measure the number of secondary infections generated from singly vs multiply infected hosts, several system-specific alternatives can be used to estimate symbiont fitness^36^. This integrative approach should aid identifying the occurrence of facilitation (e.g. see Box 2), although it may be unrealistic to apply it to (long-lived) plants. For instance, Schöb et al.^39^ highlight that identifying costs of facilitation based on measures of reproductive output during one season fails to incorporate possible compensation via higher survival in long-lived facilitators.

### Box 2 Experimental evolution of facilitation in multiple infections

Experimental evolution is a powerful method to study the evolution of facilitation (as for competition), and its consequences for symbiont virulence and transmission. This technique enables symbiont epidemiology (demography and persistence) and evolution (genetic change) to be tracked over multiple host and symbiont generations in single and multiple infections. Furthermore, facilitation occurs between many symbiont species amenable to selection (Supplementary Table 1).

**Fig. 4:**
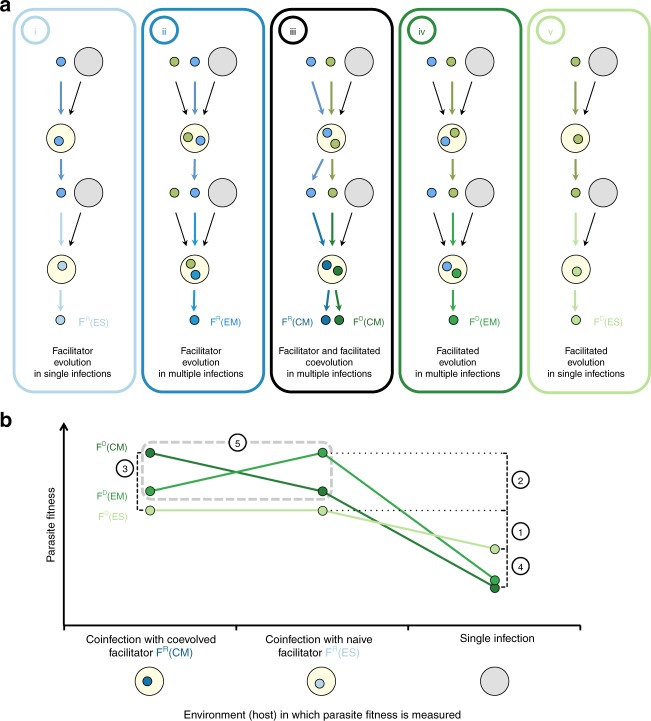
Proposed experimental design to test the evolution of facilitation. **a** Experimental design required to measure evolved responses to facilitation. Small green circles: facilitated symbionts (F^D^); small blue circles: facilitator symbionts (F^R^); big grey circles: hosts. Bold coloured arrows: (co)evolving players; thin black arrows: naive players (i.e. introduced from a naive base population at each generation). Treatments: (i) evolution of the facilitator in single infections: F^R^(ES); (ii) evolution of the facilitator in multiple infections: F^R^(EM); (iii) coevolution of the facilitated and facilitator in multiple infection: F^R^(CM) and F^D^(CM), respectively; (iv) evolution of the facilitated in multiple infections: F^D^(EM); (v) evolution of the facilitated in single infections: F^D^(ES). **b** Example of a possible evolved response of local adaptation in which facilitated symbionts have higher fitness when assayed in the infection environment in which they evolved. (1) Facilitation; (2) adaptation to the presence of a facilitator; (3) adaptation to the presence of a coevolved facilitator; (4) cost of adaptation to the facilitator; (5) local adaptation to either a coevolved or evolved facilitator

The effects of facilitation on symbiont evolution could be uncovered by allowing (i) the coevolution of both ‘facilitator’ and ‘facilitated’ symbionts in multiple infections, versus (ii) their evolution in the presence of a naive ‘facilitator’ or ‘facilitated’ (i.e. a symbiont which does not evolve in a multiply infected host), and (iii) their evolution in single infections (Figure 4). The consequences of facilitation should be assessed by measuring virulence- and transmission-related traits of evolved symbionts when tested in single and multiple infections (e.g. see ref. ^9^). However, it is not always possible to isolate different symbionts from a multiply infected host (e.g. microsymbionts or strains of the same species)^8^. Modelling approaches should thus be implemented here to elucidate, from the infection phenotype, the mechanisms at play in a particular symbiont interaction^8^. Alternatively, the specific role of different co-occurring facilitation mechanisms, such as immune- and resource-mediated, could be disentangled by manipulation of the host physiology, and by using mutant hosts lacking induced immunity to particular symbionts. Such studies might also highlight how different mechanisms, or the symmetry of the interaction (+/+, +/− or +/0) impact the evolution of facilitation.

## Ecological and evolutionary drivers of symbiont–symbiont facilitation

### Is facilitation ‘just’ ecological?

The literature on free-living organisms has identified a number of conditions favouring the occurrence of facilitation. We summarise these conditions for symbionts and draw parallels with free-living organisms in Table 1. The next question is whether these interactions persist for a sufficiently long time period, such that the facilitator may represent a selection pressure to which the facilitated can respond, and vice versa. The maintenance of, or selection for, facilitation between species thus requires that such organisms encounter one another frequently^40,41^. Factors increasing the likelihood that symbionts find themselves in the same host might be shared transmission routes^27^, or high prevalence in host populations^42^. In general, given the vast amount of evidence that has accumulated in recent decades showing that ecology and evolution operate at similar timescales (e.g. see ref. ^43^), it seems unreasonable to consider that facilitation will not be shaped by evolution. For instance, the stress-gradient hypothesis predicts the ecological conditions that favour facilitation^34^. If these conditions are constant during a given number of generations, this might select for responses in the facilitator to the facilitated, and vice versa. Similarly, it has been shown that facilitation among plants tends to occur among organisms that are more phylogenetically distant than expected by chance^7,44^. Once this interaction is established, and if sufficiently stable over time, it is likely to exert selection on the individuals involved.

**Table 1:** Factors affecting the likelihood or strength of facilitation between symbionts and parallel examples for free-living organisms

	Examples for symbiont–symbiont interactions	Examples with free-living organisms
*Intrinsic factors*		
**Developmental stage**	Interactions between microparasites in rodents can be facilitative when infections are new, but competitive when chronic (or vice versa)^40^.	The facilitative interaction between a nurse plant and a beneficiary becomes competitive as the beneficiary ages^91^.
**Genetic identity**		
Facilitated/facilitators	Facilitation only occurs between certain genotypes of spider mites on tomato plants^97^.	Only some genotypes of dinoflagellate prey are able to facilitate others by producing toxins that kill a predatory dinoflagellate^92^.
Resource/host	Facilitation between two strains of powdery mildew only occurs in more susceptible genotypes of ribwort plantain hosts^77^.	The genotype of a host plant can change the intensity of facilitative interactions occurring between beneficiaries^98^.
**Functional overlap**	In aphids, co-occuring bacterial endosymbionts often display complementary (protective and/or nutritional) functions for their hosts^41^, which increases facilitation via enhanced host fitness.	Character displacement reducing overlap in resource use between interacting free-living decomposer bacteria leads to the emergence of facilitation, as some species evolve to use the waste products of other species^48^.
**Genetic diversity**	Infection success of trematode eye-fluke parasites in rainbow trout is higher when the inoculum contains greater symbiont diversity^99^.	Species diversity of aquatic arthropods increases resource consumption compared to monospecies cultures^63^.
**Phylogenetic distance**	Facilitation occurs between both closely (e.g. two rodent malaria parasites^18^) and distantly related species (e.g. microparasites such as viruses, bacteria, fungi or protozoa, and macroparasites such as helminths^22^).	Nurse and beneficiary plants are often phylogenetically distant^7^.
*Environmental factors*		
**Demography**		
Prevalence of each player	The likelihood of facilitation is affected by the density of the facilitator (e.g. low density of a rodent malaria parasite facilitates another in mice^18^, and a high density of HIV facilitates a human malaria parasite^42^).	The strength of facilitation by the co-occurring facilitators, ribbed mussels and fiddler crabs, is positively correlated with their respective density^98^.
Order of arrival (priority effects or sequential infection)	Facilitation occurs only if the facilitator is the first to infect the host (e.g. rodent malaria parasites in mice^18^, and trematode eye-fluke genotypes in rainbow trout^99^).	Recruitment of a new grassland plant species establishing in an environment depends on the plant species that are already present^100^.
**Environmental stress**	The strength of facilitation between two strains of powdery mildew can be reduced in more resistant ribwort plantain hosts^77^.	Facilitation occurs under more stressful conditions^34^, but might disappear at the harshest end of the stress gradient (the stress-gradient hypothesis)^34^.
**Site/localisation**	The site of infection within a host determines whether facilitation occurs (e.g. scabies mites facilitate opportunistic pathogens at the wound site only^23^).	In sessile organisms, such as plants, the condition of the micro-site (soil, topography, etc) affects the intensity of the interaction among individuals^91^.

Facilitation may not only be selected following its emergence, but its emergence may be driven by evolution due to kin selection or to resolve conflicts. For instance, a novel cooperative behaviour (fibril production enhancing group migration) evolved in experimental lineages of the soil bacterium *Myxococcus xanthus*^45^. Moreover, character displacement in response to competition^46^ may decrease functional overlap between two species, which in turn could increase the likelihood of facilitative interactions^47^. For example, in free-living bacteria, facilitation can emerge due to a niche shift in response to competition for a common resource when one species evolves to exploit the waste products of another^48^. Similarly, functional overlap between bacterial endosymbionts in the lachnid aphid may have selected for function partitioning and complementation, such as between *Buchnera aphidicola* and *Serratia symbiotica* (the former has lost certain genes whose functions are encoded by the latter)^41^.

### Conditions favouring the evolution of facilitation

A main factor determining whether facilitation will evolve is the symmetry of the interaction^2,39^. In general, facilitation is predicted to be selected when the interaction is reciprocal (+/+; mutualism/cooperation), hence when the facilitator also experiences a net benefit from the interaction^2^. This is also predicted by inclusive-fitness models where cooperation between symbionts enables kin to exploit the environment and is thus selected in the presence of related individuals^49–51^. Similarly, persistent cooperation can occur between non-kin^3^ (see Box 3). For example, two different bacteriophages, in a +/+ relationship, evolved the co-packaging of their genomes for transmission to new hosts^52^. Unfortunately, this study was done with a single replicate selection line (i.e. there was no replication, as in experimental evolution replication is measured at the population/line level).

Facilitation might still evolve if it bears no cost to the facilitator (+/0; commensalism). In this case, the plant literature predicts that evolution should occur unilaterally in the facilitated species only^2^. The symbiont literature, in turn, predicts that, although facilitation is not adaptive per se, it might be indirectly selected if the trait leading to facilitation also benefits, or is genetically correlated with a trait that benefits, the facilitator^53^. For instance, facilitation via immunosuppression can be selected when it has pleiotropic beneficial effects on transmission^53^, but empirical evidence is lacking.

Finally, facilitation might evolve even when it negatively impacts the facilitator (+/−). In the plant literature, these interactions (e.g. nurse plant species negatively impacted by beneficiaries^39^) are predicted to exhibit lower evolutionary stability than mutualistic or commensal interactions, selecting for traits that eliminate associated costs; promoting tolerance or resistance to colonisation by the facilitated^2^, thereby switching the interaction towards +/0 or disappearing, respectively. Among symbionts, facilitation might be maintained if the benefit of host manipulation outweighs the cost imposed by the facilitated symbiont (see ‘Evolutionary dynamics of facilitation’ section below). Another possibility is that this type of interaction might produce an antagonistic, coevolutionary arms race between the facilitator and facilitated, as demonstrated between *Staphylococcus aureus* and *Enterococcus faecalis* over the exploitation of public goods^10^. These possibilities can be extended to plants and other free-living species, as equivalent studies are currently lacking in this area.

Moreover, interactions between symbionts and their host may also affect the probability that facilitation will evolve. Indeed, whether facilitation evolves more or less frequently among host parasites or among host mutualists remains an open question (see Box 4).

### Box 3 Intra- versus interspecific facilitation: Is there a difference?

Typically, the study of facilitation considers interspecific interactions. In contrast, positive interactions between conspecifics are studied under the umbrella of cooperation, where interactions are +/+. However, the presence of cheaters who benefit from, but do not partake in ‘common good behaviours’ are common, generating +/− or +/0 interactions^55^. A recent review^3^ highlights that within- and between species cooperation are not different evolutionary phenomena. Instead, differences between intra- and interspecific cooperation are continuous along a (phylogenetic) relatedness axis, because cooperating with an unrelated conspecific or heterospecific partner is similar. Furthermore, they discuss similarities between inter- and intraspecific cooperation across additional axes—supplied goods/services, resource competition, strategy sets and evolutionary rates—also relevant to the evolution of symbiont–symbiont facilitation^3^.

Another review^8^ addressing virulence evolution in multiple infections, discusses similarities between multi-strain versus multi-species infections. They highlight that, as a two species model^70^ assumes that interacting species are asexual, the inter and intra-specific similarities are equivalent when they interact via the same mechanism^8^. Furthermore, there is no clear theory on how the likelihood of exchanging genetic material matters to virulence^8^. Indeed, whether facilitation benefits conspecifics or heterospecifics depends mostly on the ability of individuals to exploit the modified host environment or gene product. For instance, host immunosuppression and siderophore production should benefit both conspecifics and heterospecifics^9,56,58^. Note, however, that unrelated individuals of the same or different species exploiting the common good being produced might counter-select facilitation^9,49–51^. Indeed, cooperation with kin, which increases the inclusive fitness of the facilitator, occurs only in intra-specific interactions^3^. Nevertheless, parallels might be drawn with interspecific interactions if there are phenotypic correlations between individuals partaking in a mutualism where contributing to a common good, increases the fitness of all actors^3^.

Facilitation can, however, be unique to interspecific interactions when it occurs via a mechanism or product that the facilitated symbiont cannot generate^3^. For example, when facilitation occurs via host immune trade-offs when symbiont stimulation of one branch of the immune system prevents the host from mounting an effective response against a second^21,22^. Similarly, transcapsidation or transcomplementation^15,16^, as well as nested symbionts^17^ (Fig. 3), might only apply to interspecific interactions.

### Evolutionary dynamics of facilitation

Facilitation between symbionts might not be permanent, such that the symmetry defined above (+/+, +/0 and +/−) is transitory. Consistent with this, public good models predict that cooperation should be counter-selected in the presence of non-kin^49,50^, or that facilitators should evolve to monopolise the resources they make available, thus shifting the interaction away from asymmetric facilitation^54^. However, in the latter case cheaters might also evolve to evade such monopolisation as shown in free-living systems^55^, again shifting the interaction towards asymmetric facilitation. Therefore, if players engage in arms races, facilitation might be an important force driving the evolutionary dynamics of a multiple infection even if it only occurs sporadically.

Such dynamical interactions, characterised by an arms race, might occur between herbivorous spider mite species, which make plants more suitable for themselves and competitors through down-regulation of plant defences^56–58^, but that also exclude competitors via highly localised down-regulation^57^ or the production of dense web^59^. Among plants, interactions switching between competition and facilitation due to environmental variation have been widely reported (reviewed in ref. ^11^). However, coevolutionary dynamics of facilitation between free-living organisms with asymmetric facilitation remains untested (Box 4). To our knowledge, the only example of experimental evolution of an asymmetric interaction shows facilitation loss after ten host generations^9^. Indeed, *S*. *aureus* coevolved with *E*. *faecalis* produced fewer siderophores (iron scavenging molecules) than the ancestral population and than *S*. *aureus* evolving alone^9^. This is probably because siderophores benefit *E*. *faecalis*, but the latter suppresses *S*. *aureus* growth^9^. However, corroboration of this conclusion would require experimental coevolution with mutant *E*. *faecalis* that do not benefit from siderophores (see Box 2).

### Box 4 Outstanding questions

- **Factors affecting the occurrence of facilitation between symbionts**

- Is facilitation more likely to occur between vertically transmitted symbionts or horizontally transmitted symbionts with direct transmission than between symbionts with complex life-cycles or ectosymbionts?
- Does the stress-gradient hypotheses apply to facilitation between symbionts? (e.g. How often are host switches associated with facilitation?)
- Are host condition or genetic background more important for predicting transmission than facilitation per se?

- **Factors affecting the evolution of facilitation between symbionts**

- What is the prevalence of multiply infected hosts required for facilitation to be selected?
- Is facilitation among host mutualists more likely to evolve than facilitation among parasites?

- **(Co)evolutionary dynamics of facilitation between symbionts**

- How does (co)evolving with a facilitator impact the ability of the facilitated symbiont to exploit facilitation?
- Does (co)evolution between facilitated and facilitator species select for reduced facilitation?
- Is coevolution between facilitated and facilitator species characterised by an arms race?
- Are evolved responses of the facilitator and facilitated symbionts plastic? If so, how would this impact (co)evolution between players?

- **Evolutionary consequences of facilitation between symbionts**

- Does facilitation in multiple infections select for lower or higher levels of virulence and transmission?
- Is it possible to disentangle the impact of facilitation from that of competition on virulence evolution?

- **Consequences of facilitation for disease management**

- Would efforts focussing on facilitator symbionts be a good strategy to control disease?
- How might the evolution of plastic responses in facilitator and facilitated symbionts impact control measures?

## Ecological and evolutionary consequences of facilitation

### Persistence and diversification

Facilitation between free-living organisms can increase the range distribution of a facilitated species by allowing its recruitment into environments to which it is not adapted^1^. For instance, dryland vegetation simulation models show that facilitation extends regions in which facilitated species can survive due to resource concentration by facilitator species^60^. Similarly, banded mussel patterns are predicted to permit species persistence in otherwise food scarce conditions^61^. Niche extension due to facilitation may also increase species and phylogenetic diversity^5,7^. Moreover, selection for increased facilitation is predicted to reduce extinction risk in spatially subdivided populations with local, but not global, dispersal^6^. Whether such predictions also apply to facilitation between symbionts is a timely issue and has, to our knowledge, not been addressed. Indeed, both host range expansion and parasite diversity have important consequences for disease management.

### Host health and virulence evolution

Knowledge stemming from studies of facilitation between free-living organisms could help predict the effects of facilitation between symbionts on host health. For instance, in harsh environments, facilitation can lead to the aggregation of individuals in space^62^, which, in turn, may increase ecosystem productivity compared to homogeneous distributions (e.g. mussel beds^61^ and dryland vegetation^60^). Moreover, empirical studies have shown that inter-specific facilitation can increase ecosystem functioning more generally^34,63^. This may also apply to facilitation between host mutualistic symbionts, which positively impact host health^13,47^.

These predictions from the literature on free-living organisms are, however, ignoring cases where facilitated species have negative effects on their environment. For instance, facilitation of invasive plant species (e.g. by other invasive plant species^64^, or herbivores^65^) may have deleterious consequences for ecosystem functioning reducing the biomass of resident species. Similarly, when facilitated symbionts are host parasites, laboratory observations show that facilitation often has negative impacts on host health (Supplementary Table 1). For example, a recent survey shows that multiple infections in humans can lead to higher parasite abundances and poorer host condition compared to single infections^66^. Likewise, mice infected with *Bordatella bronchiseptica* and *Heligmosoides polygyris* (see Supplementary Table 1) suffer higher mortality (accompanied by increased bacteria growth and helminth transmission) than those infected with a single species^67^. Another study shows that honey bee colony collapse might be attributed to immunosuppression caused by the mite *Varroa destructor*, promoting deformed wing virus growth, which is benign in single infections^68^.

Facilitation may also affect host health indirectly, via its effect upon virulence evolution. Facilitation is predicted to select for increased virulence by some theoretical models. Indeed, immunosuppression by one parasite might facilitate infection by a second, but subsequent competition for host resources can select for higher virulence^30^. Similarly, evolution of higher virulence is predicted in a superinfection scenario, where a first parasite facilitates infection and replacement by a second^69^ (this reduces infection duration, which selects for higher virulence). Finally, in inclusive fitness models, the production of public goods leads to elevated symbiont growth, which generally correlates positively with virulence^49^ (but see ref. ^51^).

Unfortunately, levels of virulence measured in empirical studies are not always the same as those predicted in theoretical models. Indeed, empirical studies usually measure virulence in individual hosts, within a single host generation (see examples in Table S1), whereas mathematical models analyse virulence evolution over several host generations at the population level^8^. Choisy and de Roode^70^ formalise this in a theoretical model predicting higher virulence in multiple infections that cause immunosuppression when symbionts have not evolved together, thus in the short-term. However, they also show that, in the long-term, evolution of this system selects for lower virulence, because of a longer infection duration in immunosuppressed hosts^70^. Lower virulence is also predicted to evolve when there is co-transmission of two symbionts (Box 1), one of them being a ‘helper’ required for transmission of the other^71^. This is because co-transmission, aligning the interests of co-infecting strains, decreases selection for increased within-host competition^71^.

The latter model illustrates the complex interplay between competition and facilitation that probably underlies most interactions between symbionts. As described earlier, an interaction is considered to be facilitation if its net effect is greater than that of competition. Virulence evolution will thus hinge upon the relative strength of these interactions, and the traits upon which they act. As for facilitation, competition between parasites in a multiple infection can select for higher or lower virulence^8^, or for the coexistence of strains with different levels of virulence^72^. A comprehensive understanding of virulence evolution due to facilitation thus also requires knowledge about the interplay between facilitation and competition, and underlying mechanisms. This could be investigated, for instance, in a system where the parasite life-stages engaging in each interaction are clearly defined (e.g. facilitation during the infection process followed by within-host competition for shared host resources^30^; see Boxes 2 and 4).

Furthermore, although host health is generally the read-out of virulence, it can be difficult to evaluate the relative contribution of each symbiont in multiple infections. This is easily measured when virulence and growth are correlated and when symbionts can be distinguished in a multiple infection^8^. However, virulence is not always related to symbiont growth, as assumed by most theoretical models, but might be due to immunopathology^73,74^. In such cases, symbionts in a multiple infection with facilitation might interact in such a way that virulence does not correspond to the sum of the virulence measured in each single infection. Similarly, the combined impact of host mutualists might be synergetic, additive or non-additive^47^.

Unfortunately, empirical tests of virulence evolution (i.e. across generations) in facilitating multiple infections are as yet extremely scarce (Box 2)^8^. Indeed, one study in which co-transmission evolved did not measure the virulence of the evolved viruses^52^. Others, consistent with inclusive fitness models^49–51^, found reduced virulence when bacteria producing public goods (e.g. *Bt cry* toxins produced by the Btk rif^R^ strain of *Bacillus thuringiensis*^75^, or siderophores produced by *S*. *aureus*^9^) evolved in the presence of competitors that can also exploit them (e.g. *B*. *thuringiensis* Btt spec^R^ strain, or *E*. *faecalis*, respectively). These two systems could be exploited to disentangle the relative importance of competition versus facilitation on the evolution of virulence.

## Effects on symbiont dynamics and disease management

### Transmission and epidemiology

Facilitating symbionts can enhance the transmission of (other) pathogens. For example, for super-shedding events of HIV in humans^24^, and of helminth worms in mice^67^, multiply infected individual hosts shed disproportionate numbers of transmissible stages. Moreover, transient effects of facilitation might enable a symbiont to exceed an ‘outbreak threshold’^76^, and cause epidemics even after facilitation has abated. However, true understanding of the long-term consequences of facilitation for symbiont epidemiology remains elusive.

Effects of facilitation on epidemiology have been demonstrated via long-term tracking of multiple infections in field populations. Indeed, one 5-year study, showed that the probability of infection was often higher for voles already harbouring another symbiont compared to uninfected individuals^40^. Alternatively, field observations can be corroborated with common garden experiments measuring symbiont transmission. For example, the transmission of the fungus *Podosphaera plantaginis* was higher from multiply versus singly infected *Plantago lanceolata* plants, which might explain the more severe epidemics observed in populations harbouring multiple strains^77^. Moreover, epidemiological models parameterised with empirical data show that predicted patterns coincide with field observations: positive interactions between symbionts can enhance their spread^42,78^. This approach has highlighted that reciprocal positive interactions between HIV and malaria in multiply infected hosts promote the spread of both parasites^42^, and that influenza can cause a higher incidence of pneumonia in human populations^78^. Unfortunately, theoretical models generating hypotheses on the long-term epidemiological consequences of facilitation are still scarce (but see ref. ^79^).

### Disease management

As facilitation between symbionts can promote host range expansion, parasite diversity and epidemics, exacerbate the negative effects of infection on host health and impact virulence evolution, identifying whether certain symbionts are broad-acting facilitators might, in the future, aid the development of effective control strategies. Indeed, biocontrol microorganisms^80^ might be improved if their facilitators are also introduced into risk areas. Moreover, promoting facilitation between mutualist symbionts, such as those of the gut microbiota, that increase general host health^13^ or prevent infection with parasitic symbionts^80^, could also be considered. However, such strategies should be approached with caution, as they may also entail undesirable effects. For example, facilitation of gut microbiota can lead to increased pathogenicity in normally commensal bacteria^81^. Therefore, understanding facilitation might also unveil unforeseen consequences of pest management.

One could also think about alternative strategies, aside from conventional methods of pest or parasite control, to prevent the occurrence of facilitation or facilitators, thereby controlling facilitated parasites. This might be achieved by changing the biotic or abiotic environment such that facilitation is prevented. For instance, the release of cheaters into parasite populations to counter-select strains engaged in social, facilitating behaviours linked with virulence has been suggested^82,83^. However, these strategies might be ineffective due to spatial segregation of facilitator and facilitated strains, or to phenotypic plasticity^82,83^. For instance, if phenotypic plasticity is at play, behaviours associated with facilitation and virulence (e.g. public good production) might be arrested, rather than counter-selected, in the presence of an introduced cheat^83^ (Box 4).

## Summary and outlook

### Facilitation improves understanding of host–pathogen interactions

Recent reviews highlight the importance of extending the one host—one symbiont paradigm by studying symbiont interactions in a community ecology context (e.g. see refs. ^84–86^). However, facilitative interactions among symbionts have only recently gained attention (e.g. see refs. ^27,81^), which is at odds with the broad knowledge from the plant literature. Indeed, it is clear that many open questions remain (Box 4). For instance, what conditions favour the evolution and maintenance of facilitation among symbionts (host mutualists or parasites, and/or the frequency of multiply infected hosts). And what are the longer-term consequences of facilitation? Does coevolution between facilitating and facilitated symbionts lead to reduced facilitation, or to a coevolutionary arms race? Finally, how does symbiont–symbiont facilitation affect epidemiology (transmission), and virulence evolution?

Hence, facilitation may shed light on the study of host–parasite interactions. In this sense, the extensive knowledge acquired from more than thirty years on ecological facilitation among plants may constitute a solid theoretical base to investigate the complexity of symbiont interactions and their consequences for hosts.

### Symbiont facilitation informs community ecology and evolution

Understanding facilitative interactions between symbionts might also inform on general principles in community ecology. For example, facilitation is predicted to improve ecosystem functioning^63^, to increase productivity at the community scale, and to expand the realised niche of maladapted species^1,5^. Such predictions have been corroborated by empirical studies^44^, but they would gain from being tested in manipulative experiments. As hosts are a contained environment that can harbour multiple symbiont species and can be tightly controlled and manipulated, they provide an excellent arena to establish causality, and critically test the abovementioned ideas with replication at the community level.

Symbionts can also shed new light on understanding how evolution shapes facilitation. For instance, research on facilitation among free-living organisms has ignored the possibility of an arms race between facilitators and facilitated^2,39^. Placing symbionts at the core of the facilitation literature allows not only considering, but also testing this possibility. Furthermore, given the similar timescales at which ecology and evolution operate, facilitation may be shaped by eco-evolutionary feedbacks. These may not be uncovered in other systems due to long generation times, such as long-lived plant species^39^. The typical short generation time of symbionts and their consequent amenability to experimental (co)evolution (Box 2) make them excellent candidates to address the (eco-)evolutionary consequences of facilitation at several scales.

Therefore, in the study of facilitation, as in many other areas, it is crucial to build bridges between contrasting fields of research, to generate fruitful positive feedbacks at different levels and to open new research avenues.

## Electronic supplementary material

Supplementary Material
