# Supplementary material for: Ecology and evolution of facilitation among symbionts
Source: Nat Commun. 2018 Nov 19;9:4869. doi: 10.1038/s41467-018-06779-w (PMC6242936; doi:10.1038/s41467-018-06779-w)
Supplement: Supplementary file 1 — Supplementary Material [file 41467_2018_6779_MOESM1_ESM.pdf]

Supplementary Material

Ecology and Evolution of facilitation among parasites: causes and consequences

Zélé F.[1], Magalhães S.[1], Kéfi S.[2], and Duncan A.B.[2]

1. cE3c: Centre for Ecology, Evolution, and Environmental Changes, Faculty of Sciences, University of Lisbon, Edifício C2, 38 piso, 1749-016 Lisboa, Portugal
2. Institut des Sciences de l'Évolution, Université de Montpellier, CNRS, IRD, EPHE, CC065, Place Eugène Bataillon, 34095 Montpellier Cedex 05, France
Corresponding author: Duncan A.B. (alison.duncan@umontpellier.fr)

Supplementary Table 1. Examples of selected studies that have shown facilitation between parasites. 1st column: host species full name, strains, genotypes, varieties, or breed (when applicable). 2nd and 3rd columns: "Facilitator" and "Facilitated" parasites species full names, strains, genotypes, or varieties (when applicable), and mode of transmission (H: horizontally; V: vertically; M: mixed transmission mode). 4th column: symmetry of the interaction ("+": positive; "-": negative; "0": neutral; "?": unknown effect for each partner of the interaction; 1st: facilitator; second: facilitated). 5th column: order of infection (F^R: facilitator; F^D: facilitated), and time interval between infections for sequential infections; "simultaneous": both parasites were inoculated at the same time. 6th to 8th columns: effect of facilitation on infection "success" (i.e. establishment, occurrence or prevalence), infection "intensity" (i.e. parasite load, growth, and/or development), and transmission of facilitated parasites. 9th column: consequences for the host fitness (i.e. virulence). 10th column: underlying mechanism when known; 11th column: type of study (L: laboratory; F: field; R: review; M: meta-analysis); 12th column: reference(s). Other abbreviations: ↑: increase; ↓: decrease; ⟷: not affected; "?": unkown; "-": not studied; "na": not applicable.

Host	Facilitator		Facilitated		Sym.	Infection order	success	intensity	transm.	host fitness	Mechanism	Type	Ref.
Rabbit (*Oryctolagus cuniculus*)	strongylid nematode (Graphidium strigosum)	H	strongylid nematode (*Trichostrongylus retortaeformis*)	H	-/+	na	-	↑	-	-	?	F	[1]
Nematode (*Caenorhabditis elegans ;* strain N2 wild-type)	*Staphylococcus aureus* (bacteria; strain MSSA 476)	H	*Enterococcus faecalis* (bacteria; strain OG1RF)	H	-/+	simultaneous	-	↑	-	↑ mortality	Supplied-product	L	[2]
Fruit fly (*Drosophila simulans*; strain Riverside)	*Wolbachia* (alphaproteobacteria; strain wRi)	V	Parasitoid wasp (*Leptopilina heterotoma*)	H	-/+	na	↑	-	-	↑ mortality ↓ size ↓ fecundity ↓ defences	Immunosuppression	L	[3]
spider mite (*Tetranychus piercei*)	*Wolbachia* (alphaproteobacteria)	V	*Cardinium* (flavobacterium)	V	-/+	na	-	↑	↑[a]	↑ fitness relative to uninfected hosts	Reproductive manipulation	L	[4]
Tomato leaflets (*Solanum lycopersicum*, cv Heinz)	Spider mite (*T. urticae*, 4 populations)	H	Spider mites (*Tetranychus evansi*, 2 pop.)	H	-/+	simultaneous	-	↑[b]	-	na	?	L	[5]
Tomato plant (*Solanum lycopersicum* cv Castlemart, 35S::prosystemin)	Spider mites (*T. evansi*, Algarrobo1 and Viçosa1)	H	Spider mite (*T. urticae*, Santpoort-2)	H	-/+	simultaneous; no contact	-	↑	-	↓ defence gene expression[c]	Immunosuppression	L	[6]
	Spider mites (*T. urticae*, DeLier-1)	H	Spider mite (*T. urticae*, Santpoort-2)	H	-/+	simultaneous; no contact	-	↑	-	↓ defence (late) gene expression[c]	Immunosuppression		

Host	Facilitator		Facilitated		Sym.	Infection order	success	intensity	transm.	host fitness	Mechanism	Type	Ref.
Tomato plant (*Solanum lycopersicum* cv Santa Clara- 5300)	Spider mites (*Tetranychus evansi*)	H	Spider mite (*Tetranychus urticae*)	H	-/+	F^R then F^D (0-4,7 days); no coinfection	-	↑[d]	-	↓ JA-inducible defences	Immunosuppression	L	[7,8]
Tomato plant (*Solanum lycopersicum*)	Tomato russet mite (*Aculops lycopsersici*)	H	Spider mites (*Tetranychus urticae*)	H	-/+	simultaneous	-	↑	-	↑ SA- ↓ JA-inducible defences	Immunosuppression Immune trade-off	L	[9]
Humans (*Homo sapiens*)	Human immunodeficiency virus type 1 (HIV-1)	M	Malaria parasite (*P. falciparum; protozoan)*	H	+/+	F^R then F^D (na)	↑	↑	-	↓ defences ↑ anemia ↑ postnatal mortality	Immunosuppression	R	[10]
	Malaria parasite (*P. falciparum; protozoan)*	M	Human immunodeficiency virus type 1 (HIV-1)	H		F^R then F^D (na)	-	↑	-		Immune-mediated		
Humans (*Homo sapiens*)	Herpes simplex virus (HSV2)	H	Human immunodeficiency virus type 1 (HIV-1)	M	+/+	na	?	↑	?	↑ Mucocutaneous lesions ↑ disease severity	Immune/ressource-mediated Transactivation /mechanichal	R	[11]
	Human immunodeficiency virus type 1 (HIV-1)	M	Herpes simplex virus (HSV2)	H		na	↑	↑	↑	↑ disease severity	immunosuppression		
Ex vivo lymphoid tissue (*Homo sapiens*)	Human immunodeficiency virus type 1 (HIV-1)	M	Human cytomegalovirus (HCMV)	M	+/+	F^D then F^R (overnight)	-	↑	-	-	Immunosuppression Ressource-mediated	L	[12]
Ex vivo semen (*Homo sapiens*)	Human cytomegalovirus (HCMV)	M	Human immunodeficiency virus type 1 (HIV-1)	M	+/+	unknown	-	↑	-	-	?	L	[13]
Mice (*Mus musculus*; strain BALB/c)	*Bordatella bronchiseptica* (bacteria; strain RB50)	H	Helminth (*Heligmosomoides polygyrus*)	H	+/+	simultaneous	↑	↑	↑[e]	↑ mortality	?	L	[14]
	Helminth (*Heligmosomoides polygyrus*)	H	*Bordatella bronchiseptica* (bacteria; strain RB50)	H			-	↑	-				
Mice (*Mus musculus*; strain BALB/c)	Canine influenza virus (CIV; strain H3N2)	H	*Staphylococcus pseudintermedius* (bacteria)	H	+/+	F^R then F^D (72h)	-	↑	-	↑ weight loss ↑ brain lesions ↑ lung injury	?	L	[15]
	Staphylococcus pseudintermedius (bacteria)	H	Canine influenza virus (CIV; strain H3N2)	H	+/+	F^D then F^R (72h)	-	↑	-				
Rabbit (*Oryctolagus cuniculus*)	Anoplocephaloid cestodes (Mosgovoyia pectinata)	H	Oxyurid nematode (Passalurus ambiguus)	H	+/+	na	-	↑	-	-	?	F	[1]
	Oxyurid nematode (Passalurus ambiguus)	H	Anoplocephaloid cestodes (Mosgovoyia pectinata)	H		na	-	↑	-	-	?		
Marine worm (*Olavius algarvensis*)	Sulphur-oxidizers gammaproteobacterial symbionts (γ1 and γ3)	?	Sulphate-reducers deltaproteobacterial symbionts (δ1 and δ4)	?	+/+	na	-	-	-	Allow survival[f]	Supplied-product	M	[16]
Shrimps (*Artemia franciscana* and *A. parthenogenetica*)	Cestode (*Flamingolepis liguloides*)	H	Microsporidia (*Anostracospora rigaudi* and *Enterocytospora artemiae*)	H	+/+	na	-	-	↑	na	Behavioural manipulation	F	[17]
Diverse arthropod hosts (Lepidoptera, Coleoptera, Hymenoptera and Hemiptera)	Ichneumonoid parasitoid wasps (*Braconidae* and *Ichneumonidae*)	H	Polydnaviruses (*Polydnaviridae: Bracovirus* and *Ichnovirus*)	V	+/+	na	↑	-	↑	-	Nested parasite	R	[18]
	Polydnaviruses (*Polydnaviridae: Bracovirus* and *Ichnovirus*)	H	Ichneumonoid parasitoid wasps (*Braconidae* and *Ichneumonidae*)	V	+/+	na	↑	-	-	↑ mortality	Immune-mediated		
Mosquito (*Anopheles stephensi*)	Malaria parasite (*P. chabaudi*; strain AJ; protozoan)	H	Malaria parasite (*P. chabaudi*; strain ER; protozoan)	H	+/+	F^R then F^D (4 days)	↑	↑	-	⟷ survival	?	L	[19]
	Malaria parasite (*P. chabaudi*; strain ER; protozoan)	H	Malaria parasite (*P.chabaudi*; strain AJ; protozoan)	H		F^R then F^D (4 days)	↑	↑	-	⟷ survival	?		
Spider mite (*Tetranychus phaselus*)	*Wolbachia* (alphaproteobacteria; wCon) and *Cardinium* (flavobacterium)	V	*Wolbachia* (alphaproteobacteria; wOri)	V	+/+	na	-	-	↑[a]	↓ fecundity[g] ⟷ longevity	Reproductive manipulation	L	[20]
	Wolbachia (alphaproteobacteria; wOri)	V	*Wolbachia* (alphaproteobacteria; wCon) and *Cardinium* (flavobacterium)	V	+/+	na	-	↑[h]	⟷	↓ fecundity[g] ↑ longevity	?		

Host	Facilitator		Facilitated		Sym.	Infection order	success	intensity	transm.	host fitness	Mechanism	Type	Ref.
Spider mite (*Tetranychus truncates*)	*Cardinium* (flavobacterium)	V	*Wolbachia* (alphaproteobacteria)	V	+/+	na	-	↑	↑[a]	↑ fecundity ⟷ longevity	Reproductive manipulation	L	[21]
	Wolbachia (alphaproteobacteria)	V	*Cardinium* (flavobacterium)	V	+/+	na	-	↑	↑[a]	↑ fecundity ⟷ longevity	Reproductive manipulation		
Tomato plant (*Solanum lycopersicum* cv Moneymaker)	Spider mite (*Tetranychus evansi*)	H	Spider mite (*Tetranychus evansi* or *T. ludeni*)	H	+/+	F^R then F^D (2 days); no coinfection	-	↑	-	↓ JA-inducible defences	Immunosuppression	L	[22]
	Spider mite (*Tetranychus ludeni*)	H	Spider mite (*Tetranychus evansi* or *T. ludeni*)	H	+/+	F^R then F^D (2 days); no coinfection	-	↑	-	↓ JA-inducible defences	immunosuppression		
Plantain plant (*Plantago lanceolata*; several genotypes)	Powdery mildew fungus (*Podosphaera plantaginis;* strain 10)	H	Powdery mildew fungus (*Podosphaera plantaginis;* strain 3)	H	+/+	Simultaneous	↑	↑	↑	↑ disease severity	?	LF	[23,24]
	Powdery mildew fungus (*Podosphaera plantaginis;* strain 3)	H	Powdery mildew fungus (*Podosphaera plantaginis;* strain 10)	H	+/+	Simultaneous	↑	↑	↑	↑ disease severity	?		
Pine moth (*Panolis flammea*)	Nucleopolyhedrovirus (PfNPV; Pf4)	H	Nucleopolyhedrovirus (PfNPV; Pf6)	H	0/+	simultaneous	-	↑[h]	-	↑ mortality ⟷ time to kill	?	L	[25]
Rabbit (*Oryctolagus cuniculus*)	anoplocephaloid cestode (Cittotaenia denticulata)	H	strongylid nematode (*Trichostrongylus retortaeformis*)	H	0/+	na	-	↑	-	-	?	F	[26]
Pig (*Sus scrofa domesticus*; "Large White" breed)	Scabies mite (*Sarcoptes scabiei*, var. suis)	H	Bacteria (*Staphylococcus aureus* and *Streptococcus pyogenes*)	H	?/+	F^R then F^D (<21 weeks)	↑	↑	-	↓ skin microbiota	?	L	[27]
Mice (*Mus musculus*; strain BALB/c)	Live Attenuated Influenza Vaccine (LAIV; attenuated variant of HK/Syd, HK/Sydatt/ts)	H	*Streptococcus pneumoniae* (bacteria; isolate ST425, serotype 19F)	H	?/+	both ways (7 days) or F^R then F^D (4 weeks)	-	↑	-	↑ infection duration ⟷ mortality	?	L	[28]
Mice (*Mus musculus*; strain BALB/c)	Live Attenuated Influenza Vaccine (LAIV; attenuated variant of HK/Syd, HK/Sydatt/ts)	H	*Staphylococcus aureus* (strains Wright and Newman)	H	?/+	F^R then F^D (1 and 7 days)	-	↑	-	↑ infection duration ⟷ mortality	?		
Mice (*Mus musculus*; strain BALB/cbj)	Virus Influenza A (strain H1N1; PR8)	H	*Streptococcus pneumoniae* (bacteria; strain BHN54)	H	?/+	F^R then F^D (3 days)	↑	-	-	↑ otitis media ↑ pneumonia	?	L	[29]
Mice (*Mus musculus*; strain C57Bl/6)	Virus Influenza A (strain H3N2; HKx31)	H	*Pneumococci* (serotypes 23F and 4; strains P1121 and TIGR4, respectively)	H	?/+	F^R then F^D (24h)	↑	↑	-	↑ mucus ↑ sialic acid (sugar)	Resource-mediated	L	[30]
Mice (*Mus musculus*; strain MF1)	Malaria parasite (*Plasmodium chabaudi*; protozoan)	H	Malaria parasite (*Plasmodium yoelii*; protozoan)	H	?/+	F^R then F^D (10 days)	-	↑	-	↑ mortality ↑ anemia (↓ RBC ↑ reticulocyte)	Immune-mediated Resource-mediated	L	[31]
Mice (Mus musculus; strains BALB/cByJ and C57Bl/6)	Helminth worm (*Taenia crassiceps*)	H	*Streptococcus pneumonia* (type 2 strain D39, type 3 strain A66.1, and type 19F strain BHN97)	H	?/+	F^R then F^D (8-10 weeks)	-	↑[i]	-	↑ weight loss ↑ mortality	?	L	[32]
Ferret (*Mustela putorius furo*)	Virus Influenza (strain H3N2)	H	*Streptococcus pneumoniae* (bacteria; strain BHN97)	H	?/+	F^R then F^D (3 days)	↑	-	↑	↑ disease severity	?	L	[29]
Rainbow trout (*Oncorhynchus mykiss*)	*Flavobacterium columnare* (6 different bacterial strains)	H	Trematode fluke (*Diplostomum pseudospathaceum;* 5 genotypes)	H	?/+	simultaneous	-	↑	-	↑ mortality	?	L	[33]
Rainbow trout (*Oncorhynchus mykiss*)	Trematode eye-fluke (*Diplostomum pseudospathaceum; multiple genotypes*)	H	Trematode eye-fluke (*Diplostomum pseudospathaceum; multiple genotypes*)	H	?/+	simultaneous	↑	-	-	-	?	L	[34]
Stickleback (*Gasterosteus aculeatus*)	Tapeworm (*Schistocephalus solidus*; High and Low growth populations)	H	Tapeworm (*Proteocephalus filicolis*)	H	?/+	F^R then F^D (?)	-	↑	-	-	?	F	[35]

Host	Facilitator		Facilitated		Sym.	Infection order	success	intensity	transm.	host fitness	Mechanism	Type	Ref.
Stickleback (*Gasterosteus aculeatus*)	Tapeworm (*Schistocephalus solidus*; High growth populations)	H	Trematode eye-fluke (*Diplostomum pseudospathaceum*)	H	?/+	F^R then F^D (41-43 days)	-	↑	-	-	?	F	[35]
Citrus mealybugs (*Planococcus citri*)	β-Proteobacteria	V	γ-Proteobacteria	V	?/+	na	↑	-	↑	-	Nested parasite	L	[36]
African armyworm (*Spodoptera exempta*)	*Wolbachia* (alphaproteobacteria; wExe1 and 2)	V	Nucleopolyhedrovirus (Armyworm SpexNPV)	M	?/+	na	↑	↑	-	↑ mortality	?	LF	[37]
Honeybees (*Apis mellifera*)	Parasitic mite (*Varroa destructor*)	H	Deformed wing virus (DWV)	H	?/+	F^D then F^R (unknown)	↑	↑	-	↑ mortality; (colony colapse)	Immunosuppression	F	[38]
Mosquito (*Anopheles gambiae*)	*Wolbachia* (alphaproteobacteria; wAlbB)	V	Malaria parasite (*P. berghei*; ANKA 2.34; protozoan)	H	?/+	na	⟷	↑	-	⟷ survival	?	L	[39]
Mosquito (*Culex pipiens*; strain Slab)	*Wolbachia* (alphaproteobacteria; wPip(Sl))	V	Malaria parasite (*P. relictum*; strain SGS1; protozoan)	H	?/+	na	↑	↑	-	↑ longevity<br>↓ induced mortality	?	L	[40,41]
Diverse herbivorous insect vectors and host plants	Potyviruses, waikaviruses, caulimoviruses	H	Potato aucuba virus (potyvirus) Rice tungro bacilliform virus (waikavirus)	H	?/+	na	↑	-	↑	na	Transcomplementation	R	[42–44]
Diverse plant families and aphid vectors	*Luteoviruses*	H	*Umbraviruses*	H	?/+	na	↑	-	↑	na	Transcapsidation	R	[45,44]
Mousear Cress plant (*Arabidopsis thaliana*; mutant nahG and npr1 and WT col-0)	*Pseudomonas syringae* (bacterial phytopathogen; Psm ES4326 and cfa6::Kmr)	H	Cabbage looper (*Trichoplusia ni*; inbred pop.)	H	?/+	F^R then F^D (4 days)	-	↑	-	-	?[k]	L	[46,47]
Mousear Cress plant (*Arabidopsis thaliana*; mutant npr1 and WT col-0)	*Pseudomonas syringae* (bacterial phytopathogen; strains Psm ES4326 and Pst DC3000)	H	*Pseudomonas syringae* (bacterial phytopathogen; strain Psm ES4326)	H	?/+	F^R then F^D (4 days)	-	↑	-	↓ SA-inducible defences	Immunosuppression	L	[47]
Mousear Cress plant (*Arabidopsis thaliana*; col-0)	Tomato Spotted Wilt Virus (Topsovirus ; isolate 0)	H	Western flower thrips (*Frankliniella occidentalis*)	H	?/+	Simultaneous	-	↑	-	↑ feeding scars<br>↑ SA-inducible defences	Behavioural manipulation<br>Immune trade-off	L	[48]
Pepper plant (*Capsicum annuum*)	Tomato Spotted Wilt Virus (Topsovirus)	H	Western flower thrips (*Frankliniella occidentalis*)	H	?/+	F^R then F^D (2 weeks)	-	↑	-	-	?	L	[49]
Tomato plant (*Solanum lycopersicum* cv Moneymaker)	Tomato Spotted Wilt Virus (Topsovirus ; isolate TSWV-MT2)	H	Spider mite (*Tetranychus urticae*)	H	?/+	F^R then F^D (5 weeks)	-	↑	-	↑ defences ↑ amino acid	?	L	[50]

[a] due to cytoplasmic incompatibility.
[b] population dependent effect
[c] downstream of JA and SA accumulation
[d] only in absence of coinfection: *T. urticae* is outcompeted by *T. evansi* when sharing the same leaf
[e] but no difference in the per capita (per helminth) number of eggs
[f] compensate for the loss of digestive and excretory systems (γ –bacteria produce CO2 by the oxidation of reduced inorganic compounds e.g. sulphide to sulphate. δ -bacteria reduce sulphate products produced by the γ-bacteria, producing sulphide)
[g] male effect on fecundity
[h] age-dependent effect on intensity
[i] intensity increase in the lungs only
[j] only when the host is feeding on its native host *Pinus sylvestris* but not *P. contorta*
[k] systemic response of the host

Supplementary References

1 Lello, J. *et al.* (2004) Competition and mutualism among the gut helminths of a mammalian host. *Nature* 428, 20–24

2 Ford, S.A. *et al.* (2016) Evolution of reduced pathogen virulence. *Nat. Commun.* 7, 1–9

3 Fytrou, A. *et al.* (2006) *Wolbachia* infection suppresses both host defence and parasitoid counter-defence *Wolbachia* infection suppresses both host defence and parasitoid counter-defence. *Proc. R. Soc. B* 273, 791–796

4 Zhu, L. *et al.* (2012) *Wolbachia* strengthens *Cardinium*-induced cytoplasmic incompatibility in the spider mite *Tetranychus piercei* McGregor. *Curr. Microbiol.* 65, 516–523

5 Orsucci, M. *et al.* (2016) Genotype-specific interactions between parasitic arthropods. *Heredity.* 118, 260–265

6 Alba, J.M. *et al.* (2015) Spider mites suppress tomato defenses downstream of jasmonate and salicylate independently of hormonal crosstalk. *New Phytol.* 205, 828–840

7 Sarmento, R. *et al.* (2011) A herbivorous mite down-regulates plant defence and produces web to exclude competitors. *PLoS One* 6, e23757

8 Oliveira, E.F. De *et al.* (2016) Herbivores with similar feeding modes interact through the induction of different plant responses. *Oecologia* 180, 1–10

9 Glas, J.J. *et al.* (2014) Defense suppression benefits herbivores that have a monopoly on their feeding site but can backfire within natural communities. *BMC Biol.* 12, 12–98

10 Van Geertruyden, J.P. (2014) Interactions between malaria and human immunodeficiency virus. *Clin. Microbiol. Infect.* 20, 278–285

11 Van De Perre, P. *et al.* (2008) Herpes simplex virus and HIV-1: deciphering viral synergy. *Lancet Infect. Dis.* 8, 490–497

12 Biancotto, A. *et al.* (2008) Upregulation of human cytomegalovirus by HIV type 1 in human lymphoid tissue ex vivo. *AIDS Res. Hum. Retroviruses* 24, 453–462

13 Sheth, P.M. *et al.* (2006) Disproportionately high semen shedding of HIV is associated with compartmentalized cytomegalovirus reactivation. *J. Infect. Dis.* 193, 45–48

14 Lass, S. *et al.* (2012) Generating super-shedders: co-infection increases bacterial load and egg production of a gastrointestinal helminth. *J. R. Soc. Interface* 10, 20120588

15 Kalhoro, D.H. *et al.* (2016) Canine influenza virus coinfection with *Staphylococcus pseudintermedius* enhances bacterial colonization , virus load and clinical presentation in mice. *BMC Vet. Res.* 12:87

16 Woyke, T. *et al.* (2006) Symbiosis insights through metagenomic analysis of a microbial consortium. *Nature* 443, 950–955

17 Rode, N. *et al.* (2013) Why join groups? Lessons from parasite-manipulated Artemia. *Ecol. Lett.* 16, 493–501

18 Herniou, E.A. *et al.* (2013) When parasitic wasps hijacked viruses : genomic and functional evolution of polydnaviruses. *Philos. Trans. R. Soc. B* 368, 20130051

19 Pollitt, L.C. *et al.* (2015) Existing infection facilitates establishment and density of malaria parasites in their mosquito vector. *PLoS Pathog.* 8, 1–4

20 Zhao, D. *et al.* (2013) Multiple infections with *Cardinium* and two strains of *Wolbachia* in the spider mite *Tetranychus phaselus* Ehara: revealing new forces driving the spread of *Wolbachia*. *PLoS One* 8, e54964

21 Zhao, D. *et al.* (2013) Host–symbiont interactions in spider mite *Tetranychus* truncates doubly infected with *Wolbachia* and *Cardinium*.

Environ. Entomol. 42, 445–452
22 Godinho, D.P. *et al.* (2016) Down-regulation of plant defence in a resident spider mite species and its effect upon con- and heterospecifics. *Oecologia* 180, 161–167
23 Susi, H. *et al.* (2015) Co-infection alters population dynamics of infectious disease. *Nat. Commun.* 6, 5975
24 Susi, H. *et al.* (2015) Host genotype and coinfection modify the relationship of within and between host transmission. *Am. Nat.* 186, 252–263
25 Hodgson, D.J. *et al.* (2004) Host ecology determines the relative fitness of virus genotypes in mixed-genotype nucleopolyhedrovirus infections. *J. Evol. Biol.* 17, 1018–25
26 Lello, J. *et al.* (2005) The effect of single and concomitant pathogen infections on condition and fecundity of the wild rabbit (*Oryctolagus cuniculus*). *Int. J. Parasitol.* 35, 1509–1515
27 Swe, P.M. *et al.* (2014) Scabies mites alter the skin microbiome and promote growth of opportunistic pathogens in a porcine model. *PLoS Negl. Trop. Dis.* 8, 11–14
28 Mina, M.J. *et al.* (2014) Live attenuated influenza vaccine enhances colonization of *Streptococcus pneumoniae* and *Staphylococcus aureus* in mice. *MBio* 5, 1–10
29 Mccullers, J.A. *et al.* (2010) Influenza enhances susceptibility to natural acquisition of and disease due to *Streptococcus pneumoniae* in ferrets. *J. Infect. Dis.* 202, 1287–1295
30 Siegel, S.J. *et al.* (2014) Influenza promotes pneumococcal growth during coinfection by providing host sialylated substrates as a nutrient source. *Cell Host Microbe* 16, 55–67
31 Ramiro, R.S. *et al.* (2016) Facilitation through altered resource availability in a mixed-species rodent malaria infection. *Ecol. Lett.* 19, 1041–1050
32 Apiwattanakul, N. *et al.* (2014) Chronic helminth infections impair pneumococcal vaccine responses. *Vaccine* 42, 5405–5410
33 Louhi, K.-R. *et al.* (2015) Interactions among bacterial strains and fluke genotypes shape virulence of co-infection. *Proc. R. Soc. B* 282, 20152097
34 Klemme, I. *et al.* (2016) Host infection history modifies co-infection success of multiple parasite genotypes. *J. Anim. Ecol.* 85, 591–597
35 Benesh, D.P. and Kalbe, M. (2016) Experimental parasite community ecology: intraspecific variation in a large tapeworm affects community assembly. *J. Anim. Ecol.* 85, 1004–1013
36 von Dohlen, C. *et al.* (2016) Mealybug beta-proteobacterial endosymbionts contain gamma-proteobacterial symbionts. *Nature* 412, 433–436
37 Graham, R.I. and Mushobozi, W.L. (2012) *Wolbachia* in a major African crop pest increases susceptibility to viral disease rather than protects. *Ecol. Lett.* 15, 993–100
38 Nazzi, F. *et al.* (2012) Synergistic parasite-pathogen interactions mediated by host immunity can drive the collapse of honeybee colonies. *PLoS Biol.* 8, e1002735
39 Hughes, G.L. *et al.* (2014) *Wolbachia* can enhance *Plasmodium* infection in mosquitoes: implications for malaria control ? *Plos Biol.* 10, 21–24
40 Zélé, F. *et al.* (2012) Infection with *Wolbachia* protects mosquitoes against *Plasmodium*-induced mortality in a natural system. *J. Evol. Biol.* 25, 1243–52

41 Zélé, F. *et al.* (2014) *Wolbachia* increases susceptibility to *Plasmodium* infection in a natural system. *Proc. R. Soc. B* 281, 20132837
42 Froissart, R. *et al.* (2002) Helper component-transcomplementation in the vector transmission of plant viruses. *Phytopathology* 92, 576–579
43 Pirone, T. and Blanc, S. (1996) Helper-dependent vector transmission of plant viruses. *Annu. Rev. Phytopathol.* 34, 227–247
44 Syller, J. (2012) Facilitative and antagonistic interactions between plant viruses in mixed infections. *Mol. Plant Pathol.* 13, 204–216
45 Taliansky, M.E. and Robinson, D.J. (2003) Molecular biology of umbraviruses : phantom warriors. *Jounral Gen. Virol.* 2, 1951–1960
46 Cui, J. *et al.* (2002) Signals involved in Arabidopsis resistance to *Trichoplusia ni* caterpillars induced by virulent and avirulent strains of the phytopathogen *Pseudomonas syringae*. *Plant Physiol.* 129, 551–564
47 Cui, J. *et al.* (2005) *Pseudomonas syringae* manipulates systemic plant defenses against pathogens and herbivores. *Proc. Natl. Acad. Sci.* 102, 1791–1796
48 Abe, H. *et al.* (2012) Antagonistic plant defense system regulated by phytohormones assists interactions among vector insect, thrips and a tospovirus. *Plant Cell Physiol.* 53, 204–212
49 Belliure, B. *et al.* (2005) Herbivore arthropods benefit from vectoring plant viruses. *Ecol. Lett.* 8, 70–79
50 Nachappa, P. *et al.* (2013) Tomato spotted wilt virus benefits a non-vector arthropod, *Tetranychus urticae*, by modulating different plant responses in tomato. *PLoS One* 8, e75909
